# Integration of Epigallocatechin Gallate in Gelatin Sponges Attenuates Matrix Metalloproteinase-Dependent Degradation and Increases Bone Formation

**DOI:** 10.3390/ijms20236042

**Published:** 2019-11-30

**Authors:** Anqi Huang, Yoshitomo Honda, Peiqi Li, Tomonari Tanaka, Shunsuke Baba

**Affiliations:** 1 Department of Oral Implantology, Osaka Dental University, 1-5-17, Otemae, Chuo-ku, Osaka 540-0008, Japan; huanganqident@gmail.com (A.H.); lipeiqi999@gmail.com (P.L.); baba-s@cc.osaka-dent.ac.jp (S.B.); 2 Institute of Dental Research, Osaka Dental University, 8-1, Kuzuhahanazonocho, Hirakata, Osaka 573-1121, Japan; 3 Graduate School of Science and Technology, Kyoto Institute of Technology, Matsugasaki, Sakyo-ku, Kyoto 606-8585, Japan

**Keywords:** catechin, bone formation, anti-inflammatory reaction, matrix metalloproteinase, biomaterials

## Abstract

Matrix metalloproteinase (MMP)-2 and MMP-9 are well-known gelatinases that disrupt the extracellular matrix, including gelatin. However, the advantages of modulating MMP expression in gelatin-based materials for applications in bone regenerative medicine have not been fully clarified. In this study, we examined the effects of epigallocatechin gallate (EGCG), a major polyphenol catechin isolated from green tea, on MMP expression in gelatin sponges and its association with bone formation. Four gelatin sponges with or without EGCG were prepared and implanted into bone defects for up to 4 weeks. Histological and immunohistological staining were performed. Micro-computed tomography was used to estimate the bone-forming capacity of each sponge. Our results showed that EGCG integration attenuated MMP-2 (70.6%) and -9 expression (69.1%) in the 1 week group, increased residual gelatin (118.7%), and augmented bone formation (101.8%) in the 4 weeks group in critical-sized bone defects of rat calvaria compared with vacuum-heated gelatin sponges without EGCG. Moreover, vacuum-heated gelatin sponges with EGCG showed superior bone formation compared with other sponges. The results indicated that integration of EGCG in gelatin-based materials modulated the production and activity of MMP-2 and -9 in vivo, thereby enhancing bone-forming capacity.

## 1. Introduction

Treatment of bone defects has been extensively studied in dentistry, orthopedic surgery, and plastic surgery to heal trauma, inflammation, and congenital diseases [1,2,3]. Preparation of newly formed bone is required for adequate dental implantation [3]. With advancements in tissue engineering, various biomaterials have been investigated as alternatives to autologous bone grafts, the gold standard for bone regeneration therapy, in order to overcome the need for surgical intervention and the quantitative limitations of the approach [4].

Gelatin, a denatured component of collagen, can be isolated from a variety of animals, including pigs, fish, and cows [5]. The protein is widely utilized not only in food industries but also as a biomaterial in the medical field [5]. Gelatin can be applied alone or in combination with calcium phosphates and small molecules as a scaffold for cells [6,7] and a carrier of growth factors [8] for bone tissue applications. These uses of gelatin are related to its advantageous properties, including high biocompatibility, easy procurement, and cost-effectiveness [9,10]. Meanwhile, the high biodegradability of gelatin results from enzymatic digestion or high solubility under physiological conditions, which prevent the further application of gelatin as a scaffold in bone tissue engineering [11]. To date, various studies have aimed to limit the degradability of gelatin using chemical and physical crosslinking, such as genipin, glutaraldehyde, ultraviolet irradiation, and dehydrothermal treatment [5,7,11,12]. Although these crosslinking methods indeed attenuate the degradability of gelatin, further improvements are needed to increase the bone-forming capacity of gelatin-based scaffolds comparable with autogenous bone grafts.

Matrix metalloproteinases (MMPs) are zinc-dependent endopeptidases capable of degrading extracellular matrix and were first identified in 1962 [13]. MMPs are mainly associated with physiological and pathological processes, such as inflammation, cancer, and development [14], and are regulated by reactive oxygen species (ROS) [15], inflammatory cytokines [16,17], and growth factors [18]. One family of MMPs (including MMP-2 and -9) comprises the main enzymes that function to degrade gelatin [14]. Gelatinases play key roles in the early stages of wound healing and participate in the cleavage and turnover of extracellular matrix components, including gelatin [14]. Implantation of gelatin may enhance MMP-2 and -9 expression through the foreign body reaction [19], and elevation of the expression of these gelatinases may be associated with the degradation speed of the implanted gelatin-based materials, thereby deteriorating the function of the materials in some cases.

Epigallocatechin gallate (EGCG), most abundant catechin in green tea, is widely present in foods, beverages, drugs, and biomaterials [7,20,21]. This catechin has attracted much interest owing to its numerous health benefits, including anticarcinogenic [22], anti-inflammatory [23], and anti-oxidant effects [24]. EGCG can also modulate the secretion, expression, and activation of MMPs in vitro [20,25,26,27]. Recently, our group has shown that a gelatin sponge (GS) chemically modified with EGCG (EGCG-GS) exhibits higher bone-forming capacity than gelatin containing EGCG without any chemical modification [28]. Furthermore, vacuum-heated treatment of EGCG-GS (vhEGCG-GS) significantly facilitates the bone-forming capacity of EGCG-GS in 9-mm critical-sized bone defects in rat calvaria [7]. The bone-forming capacity of vhEGCG-GS was found to be superior to that of vacuum-heated GS (vhGS). Combined use of vhEGCG-GS with multipotent progenitor cells, such as rat dedifferentiated fat cells and adipose-derived stem cells, resulted in greater bone-forming capacity than use of vhGS with both cells [29]. Based on these studies using vhEGCG-GS and vhGS, we hypothesized that postoperative modulation of MMP expression around the implanted materials may contribute to the bone-forming capacity of gelatin-based bone regenerative materials. However, little is known regarding whether modification of MMP production and activity by polyphenols directly contributes to the improvement of bone-forming capacity in gelatin-based scaffolds.

Accordingly, in this study, we aimed to confirm whether chemical modification of gelatin by EGCG enhanced bone formation through regulation of MMP expression in critical-sized defects in rat calvaria in vivo using the following four gelatin-based scaffolds: (1) GS; (2) vhGS; (3) EGCG-GS; and (4) vhEGCG-GS.

## 2. Results

### 2.1. Characterization of Sponges

**Figure 1:**
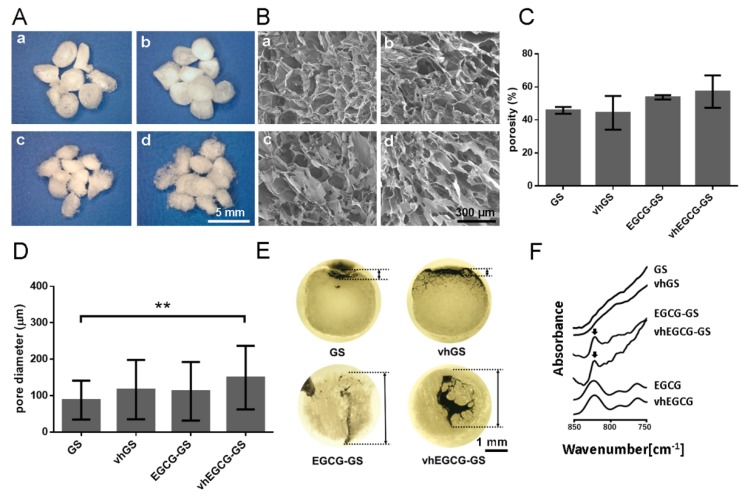
Characterization of sponges. Representative (**A**) macroscopic and (**B**) microscopic images of sponges taken by stereomicroscope and field emission-scanning electron microscope. a: gelatin sponge (GS); b: vacuum-heated gelatin sponge (vhGS); c: epigallocatechin-modified gelatin sponge (EGCG-GS); d: vacuum-heated EGCG-GS (vhEGCG-GS). (**C**) Porosity and (**D**) pore diameter of sponges. Data are the means and standard deviations (SDs). ** *p* < 0.01 (*n* = 3, ANOVA with Tukey-Kramer tests). (**E**) Ink infiltration experiment to determine the interconnectivity of pores in sponges. Bidirectional arrow: depth of ink infiltration. (**F**) Fourier-transform infrared (FTIR) spectra of sponges, intact EGCG, and vhEGCG. Arrows: EGCG peak.

Figure 1A,B show macroscopic and field emission-scanning electron microscopy (FE-SEM) images of sponges. Irregular macropores were found in all of the sponges. There were negligible differences between GS, vhGS, EGCG-GS, and vhEGCG-GS in porosity and pore size (Figure 1C,D). As for open/closed pores (interconnectivity), there was virtually no observable black ink infiltration in the GS and vhGS groups, even under deaeration conditions, while conspicuous infiltration was observed in the EGCG-GS and vhEGCG-GS groups (Figure 1E). The specific peak of EGCG was detected in the Fourier-transform infrared (FTIR) spectra of EGCG-GSs and vhEGCG-GSs, but not in those of GSs and vhGSs, suggesting that EGCG was successfully integrated into the sponges after aqueous chemical synthesis (Figure 1F).

### 2.2. Degradability of Sponges

**Figure 2:**
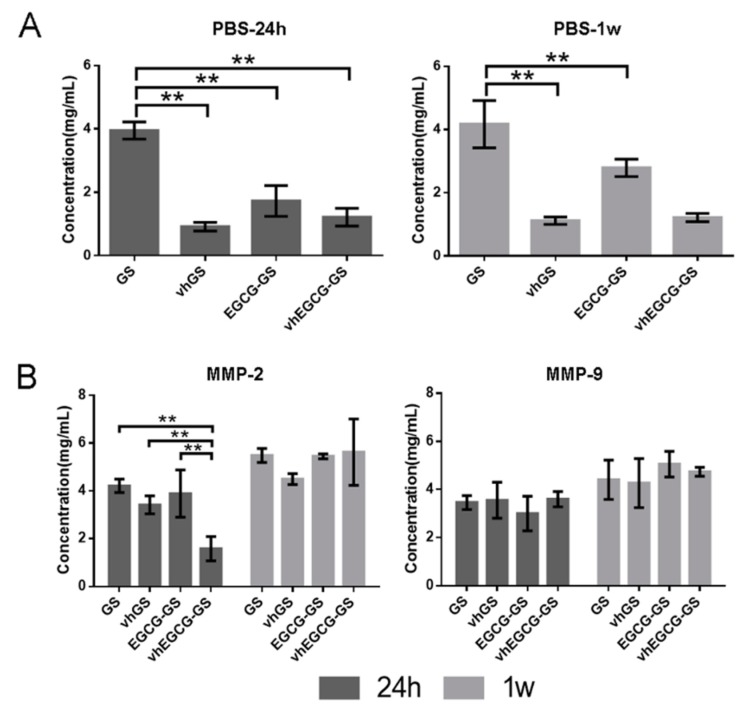
Degradability of sponges in the presence or absence of matrix metalloproteinase (MMP)-2 and -9. (**A**) Degradability of sponges in phosphate-buffered saline (PBS). (**B**) Degradation of sponges in working solution with activated MMP-2 or -9. Degradation of sponges was measured using BCA protein assays. The sponges were immersed in PBS or working solution for up to 1 week. Data are the means and SDs. ** *p* < 0.01 (*n* = 3, ANOVA with Tukey-Kramer tests).

To verify the degradability of the four types of sponges without MMPs, we immersed the sponges into phosphate-buffered saline (PBS) for up to 1 week (Figure 2A). GSs without crosslinking were immediately degraded within 24 h, whereas vhGSs and vhEGCG-GSs showed superior durability for up to 1 week. There were negligible differences between vhGSs and vhEGCG-GSs.

### 2.3. Degradability of Sponges in the Presence of MMPs

To confirm the latent degradation of sponges by MMP-2 and -9, we immersed the sponges in working solution containing activated MMP-2 and -9 in vitro (Figure 2B). Although vhEGCG-GSs significantly resisted degradation by MMP-2 for up to 24 h, all sponges were eventually degraded by both MMPs within 1 week.

### 2.4. Cell Behavior on Sponges In Vitro

**Figure 3:**
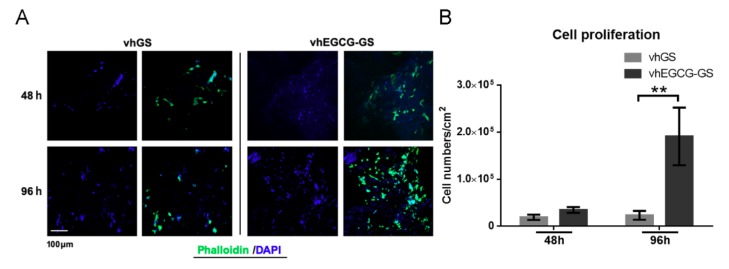
Cells grown on sponges in vitro. (**A**) Immunohistochemical images of osteoblasts (UMR-106 cells) stained with phalloidin and DAPI. Cells were seeded and cultured on the sponges for up to 96 h. (**B**) Quantitative data from (**A**). Data are means and SDs. ** *p* < 0.01 (*n* = 3, ANOVA with Tukey-Kramer tests).

Next, we evaluated the cell proliferation on vhGSs and vhEGCG-GSs using osteoblastic UMR-106 cells. The rapid disintegration of GSs and EGCG-GSs precluded the use of both sponges for the assay (Figure 2). We observed a greater extension of cells on vhEGCG-GS than on vhGSs at 96 h (Figure 3A). UMR-106 cells showed significantly better proliferation on vhEGCG-GSs than on vhGSs (Figure 3B).

### 2.5. Histological and Immunohistological Analyses

**Figure 4:**
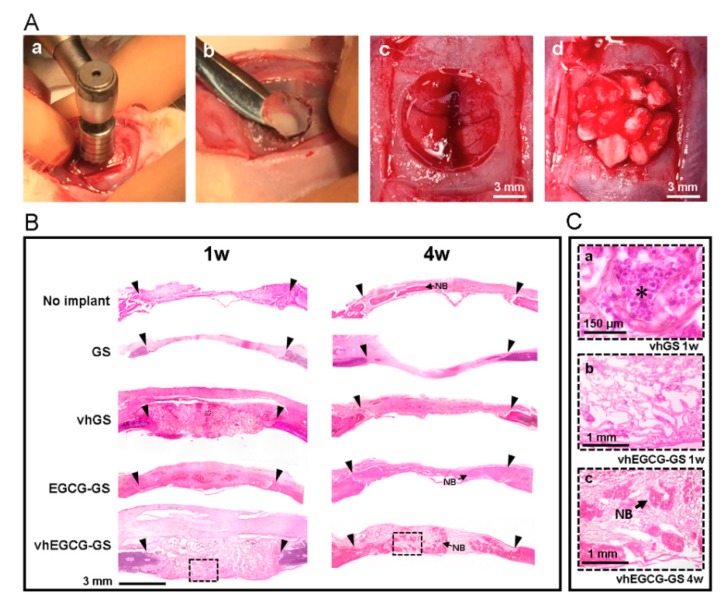
Procedure for sponge implantation and histological imaging of defects. (**A**) Work flow of the operation. a: Creation of bone defects using a trephine bar; b: removal of calvaria; c: representative critical-sized bone defects; d: defects implanted with vhGS sponges. (**B**) Low- and (**C**) high-magnification images of defects stained with hematoxylin-eosin. Outlined squares in (**B**): magnified areas in (**C**). C-a: vhGS at 1 w. C-b: vhEGCG-GS at 1 w. C-c: vhEGCG-GS at 4 w. Inverse black triangles: edges of bone defects prepared with the trephine bar. Asterisk: leukocytes. NB: newly formed bone.

**Figure 5:**
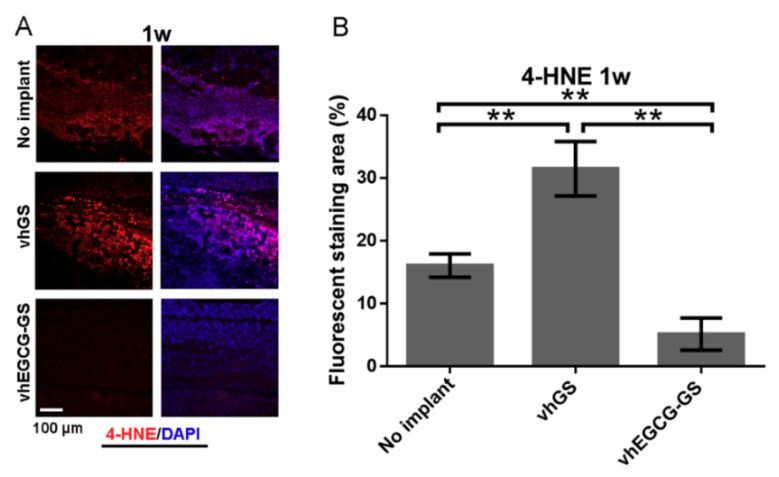
Antioxidant effects of EGCG in bone defects. (**A**) Immunohistochemical staining of defects with anti-4-hydroxynonenal (4-HNE) antibodies (red). (**B**) Fluorescently stained area of 4-HNE. (**A**) Purple: merge of 4-HNE and DAPI image. ** *p* < 0.01 (*n* = 3, ANOVA with Tukey-Kramer tests).

To confirm the biological reactions to the sponges, sponges were implanted in bone defects for up to 4 weeks (Figure 4A). Figure 4B shows hematoxylin-eosin (H-E) staining of slides of defects treated with or without sponges. Strong, widespread inflammation was observed at the defect area in the vhGS group at 1 week, whereas inflammation was reduced by 4 weeks (Figure 4(B,Ca)). Consistent with the inflammatory reactions observed in the defects transplanted with vhGSs, anti-4-hydroxynonenal (4-HNE) staining of vhGSs showed that oxidation was stronger than in the negative control (with no implantation) and in the vhEGCG-GS group (Figure 5). Thus, integration of EGCG in GSs (vhEGCG-GS) weakened 4-HNE staining at 1 week after operation in vivo.

### 2.6. MMP Expression in Defects Treated with/without Sponges

**Figure 6:**
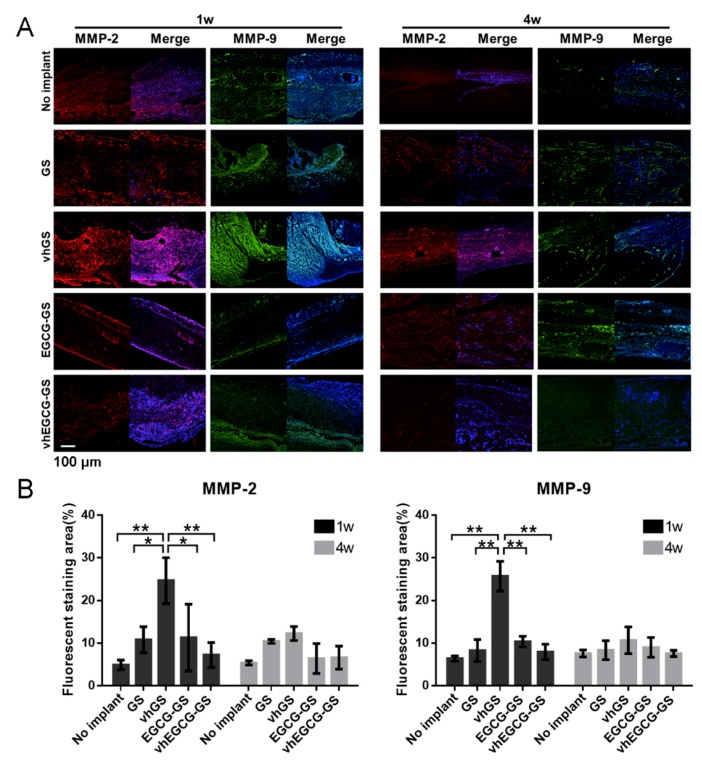
MMP-2 and -9 expression in the defects. (**A**) Immunohistochemical staining of defects treated with or without sponges for 1 or 4 weeks. Red: MMP-2; green: MMP-9; blue: DAPI; Purple: merge of MMP-2 and DAPI; light blue: merge of MMP-9 and DAPI. (**B**) Fluorescently stained area of MMP-2 or -9. Data are means and SDs. * *p* < 0.05, ** *p* < 0.01 (*n* = 3, ANOVA with Tukey-Kramer tests).

Consistent with the results of inflammatory reactions in defects treated with vhGSs, MMP-2 and -9 staining levels were higher in defects in the vhGS group compared with that in other defects at 1 week after operation (Figure 6). Moreover, chemical modification of EGCG on gelatin (vhEGCG-GS) attenuated MMP expression at 1 week to a level similar that in the no implant group.

### 2.7. Bone-forming Capability of Sponges

**Figure 7:**
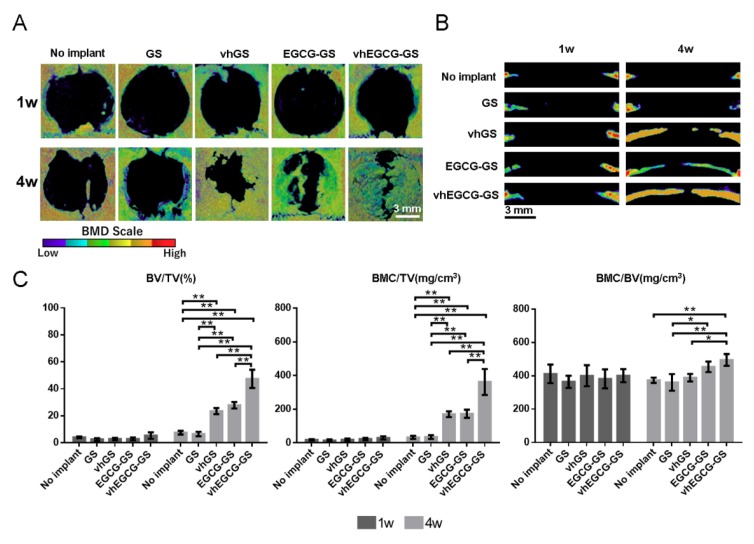
Morphometric analysis of defects using microcomputed tomography (μCT). Vertical (**A**) and lateral (**B**) bone mineral density (BMD) images of defects treated with or without sponges for 1 or 4 weeks. (**C**) Morphometric analysis of bone defects. BV: bone volume; TV: total volume; BMC: bone mineral content. Data are means and SDs. ** *p* < 0.01 (*n* = 3, ANOVA with Tukey-Kramer tests).

Histological and morphometrical analyses of bone formation in defects were performed using H-E staining and micro-computed tomography (μCT) analysis (Figure 4 and Figure 7). The radio-opacities of all defects increased over time, even in the no implant group (Figure 7). We confirmed that those radio-opacities were consistent with the newly formed bone using H-E staining (Figure 4(B, Cb Cc)). vhEGCG-GSs showed superior bone-forming capacity, as estimated using bone volume/total volume (BV/TV) and bone mineral content (BMC)/TV, compared with those of other sponges and without implantation. When compared to GSs and vhGSs, chemical modification of gelatin by EGCG (EGCG-GSs and vhEGCG-GSs) enhanced bone formation at 4 weeks after operation. There were negligible differences in BMC/BV (as a measure of bone quality) in newly formed bone within the defects among all groups.

### 2.8. Residual Sponges in the Defects

**Figure 8:**
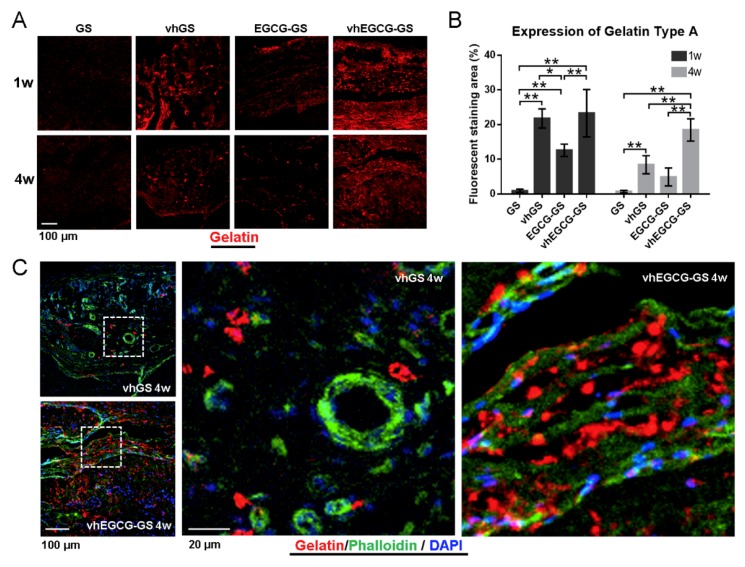
Residual gelatin in defects treated with sponges. (**A**,**C**) Immunohistological staining of type A gelatin for 1 or 4 weeks after implantation. Red: gelatin; green: phalloidin; blue: DAPI. (**B**) Fluorescently stained area of gelatin at (**A**). Data are means and SDs. * *p* < 0.05, ** *p* < 0.01 (*n* = 4, ANOVA with Tukey-Kramer tests). (**C**) Actin staining of cells using phalloidin for defects treated with vhGSs and vhEGCG-GSs 4 weeks after implantation. Outlined squares: magnified area.

Figure 8 shows immunohistochemical images of residual gelatin in defects treated with or without sponges. vhEGCG-GSs retained more gelatin in defects for up to 4 weeks compared with GSs, vhGSs, and EGCG-GSs (Figure 8A,B). Phalloidin staining revealed that the cells were distributed along the gelatin within vhEGCG-GSs, whereas cells were sporadically distributed in defects treated with vhGSs (Figure 8C).

## 3. Discussion

In the current study, we showed that the presence of chemically modified EGCG in gelatin (vhEGCG-GSs) markedly reduced MMP-2, MMP-9, and 4-HNE levels in defects compared with that in samples treated with gelatin without EGCG (vhGS). Osteogenesis induced by vhEGCG-GSs was greater than that induced by vhGS lacking EGCG integration. Residual gelatin in vhEGCG-GSs established an appropriate, robust framework for cells and could facilitate cell proliferation, differentiation, and migration during osteogenesis. Overall, the results indicated that modification of MMP-2 and -9 expression by chemical modification of gelatin with EGCG could augment the bone-forming capacity of gelatin-based materials by providing an appropriate scaffold for cell adhesion.

It is widely recognized that differences in porosity, pore size, and interconnectivity can modify the bone-forming ability of biomaterials [30]. Our results show that there were negligible differences among the four samples in terms of porosity and pore size, while there were obvious differences in interconnectivity between sponges with and without EGCG (Figure 1C–E). The results of μCT analysis demonstrated that the EGCG-GS and vhEGCG-GS groups also showed better bone-forming capabilities than the GS and vhGS groups (Figure 7). Interestingly, the vhEGCG-GS and EGCG-GS groups showed a significant difference in bone formation, even though these materials had similar levels of interconnectivity. Thus, while interconnectivity may be partially associated with the bone-forming ability of sponges, it appears not be a critical factor in the remarkable bone formation ability of vhEGCG-GS.

MMP-2 and -9 degrade gelatin as their main substrate [14], although the ability of these proteinases to degrade vhGSs and vhEGCG-GSs has not been evaluated. In comparison with the degradation speed of GSs in PBS, hydrothermal treatment by vacuum heating at 150 °C, causing physical crosslinking, increased the robustness of both GSs and EGCG-GSs. However, vhGSs could still be degraded after exposure to a sufficient amount of MMP-2 and -9 in vitro, indicating that physical crosslinking induced by dehydrothermal treatment of gelatin-based materials was not always sufficient to provide complete enzymatic resistance against MMPs.

At 1 week after implantation of the sponges, the treatment of defects with vhGSs temporally enhanced early inflammation, as demonstrated by the presence of leukocytes in sponges, and augmented 4-HNE staining as a measure of oxidation. Defects treated with vhEGCG-GSs showed reduced leukocyte recruitment and weaker 4-HNE staining than defects treated with vhGSs. A previous study showed that ROS indirectly facilitate the expression and activation of MMPs [15]. EGCG is a well-known antioxidant [31], and we previously reported that gelatin solutions chemically modified with EGCG decreased intracellular ROS levels in macrophage cell lines [24]. These results suggested that the EGCG in vhEGCG-GSs could suppress ROS production, thereby partially inhibiting the expression of MMPs.

Excess MMP-2 and -9 degraded vhGSs within 24 h. Although we could not identify the exact concentrations of MMP-2 and -9 at the site of bone defects in vivo, our results revealed that chemical modification of EGCG on gelatin delayed the degradation of gelatin on vhEGCG-GSs in the presence of MMP-2 in vitro. Previous in silico analyses revealed that EGCG has inhibitory effects on MMP-2 via strong interactions [32]. Thus, integration of EGCG in vhEGCG-GSs may not only decrease the expression of MMPs but also contribute to counteracting the enzymatic reaction by MMPs at bone defects in vivo.

The osteogenesis induced by vhEGCG-GSs showed the opposite pattern as MMP-2 and -9 staining, but similar pattern relative to the quantity of residual gelatin. These tendencies revealed that residual vhEGCG-GSs could provide scaffolds for cells to induce bone formation. Indeed, in our immunohistological staining of actin using phalloidin, we found that cells were distributed along the residual gelatin of vhEGCG-GSs at 4 weeks, and sporadic cells were present at bone defect areas in the vhGS group. The attached cells, similar to osteoblasts and multipotent progenitor cells associated with bone formation, require robust cell attachment for survival [33,34]. Rapid digestion of gelatin by MMP-2 and -9 could yield a fragile, brittle scaffold for cell attachment and prevent bone formation by vhGSs, whereas vhEGCG-GSs seemed to provide a sufficient scaffold for cells via the inhibitory effects of MMPs.

Although our data demonstrated that chemical modification of gelatin by EGCG mitigated MMP-2 and -9 expression in vivo, further experiments are required to verify the detailed mechanisms underlying the relationships between the control of MMP expression and the bone-forming capacity of GSs. For example, the types of cells that secrete MMP-2 and -9 around the GSs have not been identified, and no studies have demonstrated how EGCG in vhEGCG-GSs hampers the expression of MMPs by these cells. Additionally, it is unclear whether bound or unbound EGCG can attenuate the expression and activation of MMP-2 and -9 in vivo. It would also be beneficial to investigate the effect of chemical modification of EGCG for other gelatin-based materials. However, our data provided evidence that integration of certain polyphenols as anti-MMP reagents could be a useful strategy for decreasing the biodegradability of GSs, a critical drawback of gelatin use in scaffolds in vivo.

## 4. Materials and Methods

### 4.1. Preparation and Characterization of Sponges

EGCG and porcine type A gelatin were purchased from the Bio Verde Inc. (Kyoto, Japan) and Sigma-Aldrich (St. Louis, MO, USA). 4-(4,6-Dimethoxy-1,3,5-triazin-2-yl)-4-methylmorpholinium chloride (DMT-MM) and N-methylmorpholine (NMM) were purchased from Tokyo Chemical Industry Co. Ltd. (Tokyo, Japan) and Nacalai Tesque Inc. (Kyoto, Japan), respectively. EGCG-GS was prepared as reported previously [7,28]. Briefly, 100 mg gelatin, 0.07 mg EGCG, 69.2 mg DMT-MM, and 27.5 μL NMM were dissolved and stirred in 5 mL MilliQ water at room temperature. This EGCG dose was determined based on our previous report [7]. After complete reaction of each reagent, residual reagents were removed by the dialysis procedure using Spectra/Por7 MWCO 1000 (Spectrum Labs, Rancho Dominguez, CA, USA) in water in the dark. The resulting solution was diluted up to 10 mL with MilliQ water and frozen at −30 °C, followed by lyophilization using DC800 (Yamato Co., Ltd., Tokyo, Japan) in φ5-mm silicon tubes. vhEGCG-GS was prepared by heating EGCG-GS with an ETTAS AVO-250NS (AS ONE, Osaka, Japan) at 150 °C for 24 h with a gauge pressure of −0.1 MPa. These conditions (without EGCG, DMT-MM, and NMM) were used to prepared GSs and vhGSs as a control. Macroscopic and field emission-scanning electron microscopic images were obtained using stereomicroscopy (SZX12; Olympus Inc., Tokyo, Japan) and FE-SEM (S-4800; Hitachi, Tokyo, Japan). The average pore size and porosity were determined from FE-SEM images using an image analysis program (ImageJ version 1.50i, Bethesda, MD, USA) following slight modification of a method reported previously [35,36]. Fifty pores in each material were measured to determine the pore diameter, which was defined as the maximum diameter of each pore. Porosity was analyzed in three pictures from each group. To evaluate the interconnectivity, 1 μL black ink was dripped onto each sponge material. The samples were vacuumed for 30 min and dried at 60 °C for 30 min. Then, the sponges were cut, and the cross-sections were observed using a stereomicroscope (SZX12; Olympus Inc.). To evaluate the presence of EGCG, Fourier-transform infrared spectroscopy analysis was performed using a Spectrum One instrument (PerkinElmer, Inc., Waltham, MA, USA). The sponges were evaluated over a range of 850 to 750 cm^−1^ with 2 cm^−1^ resolution.

### 4.2. Degradation Assays

Degradation assays were performed to analyze whether the sponges were degradable under conditions with or without MMP-2 and -9. Two-milligram sponges were incubated in 300 μL PBS or working solution with or without both MMPs at 37 °C for 24 h or for 1 week at 60 rpm using a constant temperature incubator (Taitec BR-40LF; Taitec Co., Ltd., Saitama, Japan). Working solution was composed of 30 μL of 10% Triton X-100, 270 μL PBS, and 0.5 μg MMP-2 or -9 protein powder (MMP-2: cat. No. GTX 48396-Pro; MMP-9: cat. No. GTX 65181-Pro; GeneTex, Inc., Irvine, CA, USA). After incubation, 10 μL supernatant was extracted, and total protein was assayed using a Protein Assay BCA Kit (Thermo Fisher Scientific Inc., Waltham, MA, USA).

### 4.3. Cell Maintenance and Staining In Vitro

The osteoblastic cell line UMR-106 (cat. No. CRL-1661; American Type Culture Collection, Manassas, VA, USA) was used to evaluate the behaviors of osteoblasts [37]. UMR-106 cells were maintained in Dulbecco’s modified Eagle’s medium high-glucose (DMEM; Sigma-Aldrich Co.) with 10% fetal bovine serum (FBS) and 1% antibiotics in a 5% CO_2_ incubator at 37 °C. The cells were injected into the sponges (3 mm in diameter × 10 mm in height) at 3 × 10^4^ cells/sponge and cultured in 24-well plates for 48 or 96 h in DMEM with 10% FBS and 1% antibiotics. At the appropriate time, cells were stained with Alexa Fluor 488 phalloidin (Thermo Fisher Scientific Inc.; 1:200). Samples were mounted using DAPI Fluoromount-G (SouthernBiotech, Birmingham, AL, USA) and analyzed using a ZEISS LSM700 microscope (Carl Zeiss Microscopy, Jena, Germany). Cell numbers were counted using NIH ImageJ version 1.50i (Bethesda) in the particle analyzer mode. Cell numbers were calculated as the cell number/cm^2^.

### 4.4. Animal Experiments

The animal experiments in this study strictly followed the guidelines approved by the local ethics committee of Osaka Dental University (approval no. 18-02003, approved on 3/15/2018). Male Sprague-Dawley rats (8 weeks old) were as purchased from Shimizu Laboratory Supplies Co. (Kyoto, Japan). The rats were anesthetized by intraperitoneal injection of a mixture of medetomidine hydrochloride (0.15 mg/kg; Domitor; Zenoaq, Fukushima, Japan), midazolam (2 mg/kg; Midazolam Sandoz, Sandoz K.K., Yamagata, Japan), and butorphanol tartrate (2.5 mg/kg; Vetorphale, Meiji Sika Parma Co., Ltd., Tokyo, Japan). Critical-sized bone defects (9 mm in diameter) were prepared at the center of calvaria, as reported previously, using a trephine bar [38]. Rats in the negative control group did not receive implantation. Sponges were implanted in the defects for the experimental groups. Approximately ten sponges (each sponge measured approximately 3 mm in diameter × 3 mm in height) were implanted in each defect. Three rats in each group were used for the experiments shown in Figure 4, Figure 5, Figure 6 and Figure 7, while four rats per group were used for that shown in Figure 8 (additional experiments were performed to provide enough samples for immunohistochemical staining). At 1–4 weeks after implantation, rats were euthanized with isoflurane, and calvaria were fixed with 4% paraformaldehyde in 0.1 M phosphate buffer for subsequent experiments.

### 4.5. Histological and Immunohistological Analyses

After μCT scanning, nondecalcified cryosections were obtained using the Kawamoto method [39] and a cryotome (Leica CM3050S; Leica Biosystems Inc., Buffalo Grove, IL, USA). Sections were stained with H-E to confirm newly formed bone and the early inflammatory reaction, with 4-HNE antibodies for detecting the antioxidant effects of EGCG, anti-MMP-2 and -9 antibodies for evaluating MMP expression, and anti-gelatin antibodies for estimating residual gelatin. For immunohistological analysis, tissue sections were immunolabeled with the following primary antibodies: rabbit anti-rat MMP-2 polyclonal antibodies (cat. No. 10373-2-AP; Proteintech Group, IncRosemont; 1:500 in PBS), rabbit anti-rat MMP-9 polyclonal antibodies (cat. No. 10375-2-AP; Proteintech Group; 1:300 in PBS), anti-gelatin type A antibodies (cat. No. GELP-12-N-10; Alpha Diagnostic Intl., Inc. San Antonio, TX, USA; 1:200). After washing with PBS, the sections were stained with the following secondary antibodies: Alexa Fluor 594 goat anti-mouse IgG (Thermo Fisher Scientific Inc., Waltham, MA, USA; 1:200) for MMPs and Alexa Fluor 647 goat anti-mouse IgG (Thermo Fisher Scientific Inc.; 1:200) for gelatin. Rabbit anti-4-HNE polyclonal antibodies conjugated with Alexa Fluor 647 (cat. No. bs-6313r-a647; Bioss Inc., MA, USA; 1:200) were used to detect oxidation in the defects. Cells aligned to the gelatin were detected using Alexa Fluor 488 phalloidin (Thermo Fisher Scientific Inc.; 1:200). The tissue slides were mounted in DAPI Fluoromount-G (SouthernBiotech) and analyzed with a ZEISS LSM700. Quantitative calculation of fluorescence images was performed using NIH ImageJ version 1.50i. A region of interest (ROI) was randomly selected from each section. The percentage of fluorescent area in the defect was calculated as follows: (fluorescent area/total tissue area in ROI) × 100.

### 4.6. μCT Analysis

To evaluate the bone-forming capacity of sponges and the quality of newly formed bone, harvested calvaria implanted with or without sponges were assessed using μCT (SMX-130CT; Shimadzu, Kyoto, Japan). Samples were scanned with a 55-kV tube voltage and a 90-μA tube current. Images were saved at a resolution of 512 × 512 pixels. Bone mineral density images of samples were vertically and laterally reconstructed using a three-dimensional (3D) image analysis system (TRI/3D-Bon; Ratoc System Engineering, Tokyo, Japan). Bone formation was estimated from BV/TV and BMC/TV, whereas the quality of newly formed bone was estimated from BMC/BV. BMC, which represented calcified bone tissue, was quantified using cylindrical phantoms containing hydroxyapatite (200–1550 mg/cm^3^).

### 4.7. Statistical Evaluation

Statistical significance was calculated using one-way analysis of variance, followed by Tukey-Kramer tests. Microsoft Excel software and an add-in statistical package (Statcel 4; OMS, Saitama, Japan) were utilized for the calculations. All experiments except for the animal experiments were performed with at least two independent replicates, and all results showed high reproducibility.

## 5. Conclusions

In the current study, we found that chemical modification of gelatin by EGCG blocked the early expression of 4-HNE (a marker of oxidation), MMP-2, and MMP-9; thus, more residual gelatin was retained in defects treated with vhEGCG-GSs until 4 weeks after implantation. Consistent with this finding, osteogenesis by vhEGCG-GSs was significantly superior to that induced by vhGSs or no implantation, possibly because the residual gelatin functioned as an effective scaffold for osteoblasts and multipotent progenitor cells. Our study established a model for regulation of MMP production, and our results provided important insights into the development of promising gelatin-based biomaterials for bone tissue regenerative medicine.

## Abbreviations

GS	Gelatin sponge
vhGS	Vacuum heated gelatin sponge
EGCG	Epigallocatechin gallate
EGCG-GS	Epigallocatechin gallate-modified gelatin sponge
vhEGCG-GS	Vacuum heated EGCG-GS
